# Revealing the hidden harms in end-of-life care: a mixed-methods characterisation of reported safety incidents involving injectable symptom control medication

**DOI:** 10.3399/BJGP.2025.0301

**Published:** 2026-01-27

**Authors:** Isabel Hope, Ben Bowers, Isobel J McFadzean, Sarah Yardley, Kristian Pollock, Stuart Hellard, Andrew Carson-Stevens

**Affiliations:** 1 Patient Safety Research Group, Division of Population Medicine, School of Medicine, Cardiff University, Cardiff, UK; 2 Marie Curie Research Centre, Division of Population Medicine, School of Medicine, Cardiff University, Cardiff, UK; 3 Department of Public Health and Primary Care, University of Cambridge, Cambridge, UK; 4 School of Health Sciences, University of Nottingham, Nottingham, UK; 5 Queen's Nursing Institute, London, UK; 6 Marie Curie Palliative Care Research Department, Division of Psychiatry, University College London, London, UK; 7 PRIME Centre Wales, Division of Population Medicine, School of Medicine, Cardiff University, Cardiff, UK

**Keywords:** caregivers, palliative care, patient safety, primary health care, safety, subcutaneous

## Abstract

**Background:**

Many patients dying in the community are prescribed injectable medications and are vulnerable to unsafe care. Developing safer and effective healthcare systems requires learning from patient safety incidents, including those resulting in no harm or near misses, however, health systems typically, must prioritise learning from harmful incidents because of resource constraints and are at risk of missing key learning.

**Aim:**

To appraise the nature and outcomes of reported ‘no harm’ injectable end-of-life symptom control medication incidents, and understand the characteristics of those incidents and how they differ from those reclassified during the study analysis as ‘harmful’.

**Design and setting:**

This was a mixed-methods analysis of nationally reported (England and Wales) patient safety incidents to the National Reporting and Learning System involving injectable end-of-life symptom control medications in the community.

**Method:**

A random sample of 1000 incidents reported as ‘no harm’ incidents submitted between 2017 and 2022 was screened. The PatIent SAfety (PISA) classification system was used to characterise incident type, contributory factors, reported harms, and outcomes, with subsequent thematic analysis of free-text narratives.

**Results:**

In total, 388 incidents were included. Of these, 107 (28%) reports described harm to patients and families including 43 that detailed psychological harms. Comparing incidents reclassified as harmful with the true ‘no harm’ incidents, the harmful incidents contained more conflicting views between professionals and family members and there was clear variability in perceptions of what constitutes a harm.

**Conclusion:**

Healthcare teams need to incorporate the impact on the patient and families when reporting and learning from end-of-life symptom control incidents, notably resultant emotional and psychological harms.

## How this fits in

Public inquires have raised serious concerns about the use and mismanagement of injectable end-of-life symptom control medications. National-level patient safety data remains underutilised for learning from reported incidents and near misses involving these medications. This study analysed the outcomes and nature of harms reported as ‘no harm’ incidents and identified that 28% (107/388) of these free-text narratives detailed physical harms and/or psychological harms. Organisations should empower their teams to report and learn from ‘no harm’ incidents and ensure their definitions of harm are inclusive of psychological harm and their staff recognise these forms of harm.

## Introduction

GPs and community nurses lead and provide most end-of-life care, even if patients subsequently die in hospital.^1,2^ As patients deteriorate, they can struggle to take oral medications, so subcutaneous medications are often prescribed ahead of need (anticipatory medications) or in response to worsening symptoms.^3^ These injectable medications are usually prescribed for the symptoms of pain, breathlessness, nausea, vomiting, agitation, and respiratory tract secretions.^4^ Between 40% and 51% of patients dying in the community are prescribed injectable anticipatory medications in the UK.^4–6^ This practice varies internationally, with Canada and Australia adopting similar guidance to the UK.^7,8^ Despite being widespread and recommended practice,^9,10^ there is insufficient evidence regarding the impact of injectable medications on patient safety, clinical effectiveness, and experiences of care.^5,11^ Two recent UK independent reviews raised serious concerns about the mismanagement of injectable end-of-life symptom control medications,^12,13^ suggesting medications were being inappropriately prescribed and administered, often without sufficient communication with patients or families.

Patient safety is a healthcare priority internationally^14^ and is defined as the avoidance of unintended or unexpected harm to people during the provision of health care.^15^ Patient safety incident reporting and learning systems have been established in many countries to aid the capture of learning to help care providers understand the context and contributory factors influencing patient safety events. Such learning can be used to understand where the systems can be improved for safe care for future patients.^14^ In the NHS, healthcare teams are expected to report such incidents through an electronic template that is reviewed locally by managers. At an organisation level, patterns in reported incidents might initiate local investigations and, at a national level, commonalities in rarer incidents occurring at a local level can be identified and raise recommendations for improvements at a policy-, manufacturing-, and practice-level.^16^

Over the past two decades, it has become widely accepted that the patient safety incidents that are interjected by a healthcare professional, patient or family member (so called ‘near misses’) as well as the incidents resulting in ‘no harm’ overall are likely to contain the same contributory factors as their harmful counterparts.^17^ This perspective offers healthcare systems an opportunity to shift their strategy from retrospective patient safety incident management to prospective proactive risk management.^18^ In this paper, near misses and no harm incidents will be the focus of this study and will be referred to collectively as ‘no harm incidents’; such incidents occur far more frequently than harmful incidents^19^ and systems thinkers highlight their potential to offer valuable insights into what can be improved in complex systems.^16^ However, current healthcare systems are often constrained by the challenges of processing high volumes of reports with limited resources, so reported no harm incidents are less likely to gain attention and stimulate system-wide learning.^20,21^

Half of avoidable healthcare harms internationally involve medications.^22,23^ Dying patients are particularly vulnerable to medication-related harms^24,25^ and have reduced resilience to unsafe care.^26^ Direct physical harms to patients have taken precedent in health care (for example, prolonged pain); however, within palliative care, there is an emphasis on holistic care encompassing physical, psychological, social, and spiritual support.^27,28^ Therefore, potential harms are not limited to direct physical harm but encompass psychological, social, and spiritual domains for patients plus their families and carers.^29^ Two recent studies indicated that ‘no harm’ palliative care medication incident report narratives detailed a range of physical and psychological distress to patients and families.^24,30^ The nature of these underrecognised harms warrants closer, systematic investigation.

Our research aims were to appraise the nature and outcomes of reported ‘no harm’ injectable end-of-life symptom control medication incident narratives in community care settings and understand the characteristics of reported ‘no harm’ incidents and how they differ from those reclassified during the study analysis as ‘harmful’.

## Method

A mixed-methods analysis of nationally reported injectable medication-related patient safety incidents was conducted. The descriptive quantitative analysis informed the subsequent qualitative analysis.^31^

The study team comprised an academic GP registrar (joint first author), a clinical academic community nurse (joint first author); two academic GPs (third author and senior author); a medical sociologist (fifth author); an academic palliative medicine consultant (fourth author), and a data manager (sixth author).

### Data source

Recorded incidents were sourced from the National Reporting and Learning System (NRLS) database for England and Wales, the largest incident database worldwide. NRLS was superseded in 2023 by the Learn from Patient Safety Events service. Patient safety incidents are defined as: ‘something unexpected or unintended has happened, or failed to happen, that could have or did lead to patient harm.’^32^ No harm and near miss incidents are categorised as ‘no harm’ incidents within the NRLS database. Reports contain structured information about the incident and free-text narratives describing what happened and why, and actions to prevent reoccurrence.

### Study population, setting, and sample

A systematic search of all NRLS incident reports for adult patients occurring in community settings between 30 April 2017 and 30 April 2022 was conducted. To identify relevant reports, free-text fields were systematically searched using keywords for palliative care and injectable end-of-life symptom control medications (Supplementary Information S1). The search strategy built on previous successful methods.^16,33^

The search returned 20 958 ‘no harm’ incidents and a random sample of 1000 was used. This sample size enabled us to conduct an exploratory mixed-methods study within the resources available. These were manually screened using the study inclusion and exclusion criteria (Box 1). The first 200 reports were screened independently by the joint first authors, with excellent concordance (0.95 Cohen’s Kappa). Consensus decisions informed one of the joint author’s subsequent screening (the academic GP registrar), with the other joint author (the clinical academic community nurse) and the third author providing review in cases of uncertainty.

**Box 1.** Study inclusion and exclusion criteria

Inclusion criteria	Exclusion criteria
The report met the definition of a patient safety incident or near miss^32^ Incident involved injectable end-of-life symptom control medications Patient in last phase of life: report indicates that receiving (or should receive) palliative and end-of-life care Incident occurred in community settings, which included transitions between care settings Patient aged ≥18 years	Report did not involve a patient safety incident or near miss Injectable medications not involved in incident Injectable medication used without palliative intent Incident occurred solely within an inpatient care setting: hospital, hospice, mental health, and learning disability inpatient units

### Descriptive analysis

**Figure 1.**
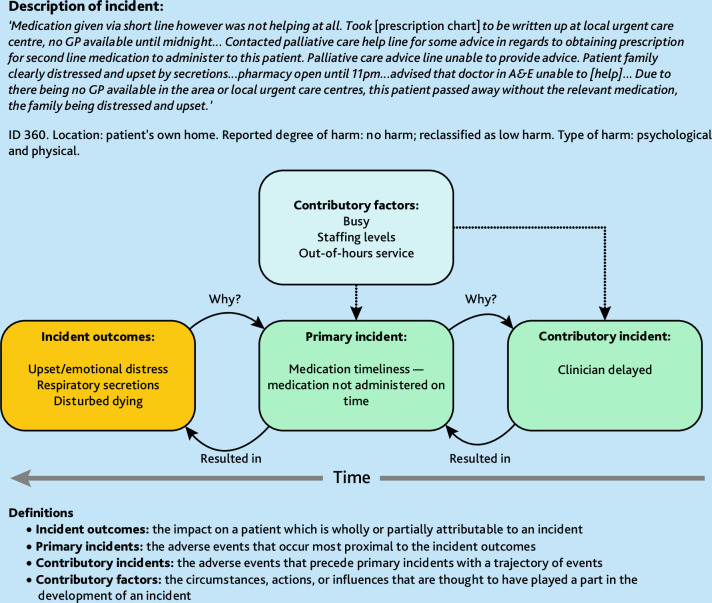
Recursive model of incident analysis and definitions. A&E = accident and emergency.

The PatIent SAfety classification (PISA) frameworks were used by one of the joint authors (the academic GP registrar) to deductively code eligible incident report narratives.^16^ PISA is comprised of integrated coding frameworks that have been empirically developed from the analysis of >120 000 patient safety incident reports, drawing on the World Health Organization International Classification for Patient Safety ontology.^34^ This recursive model of incident analysis was used to deconstruct incident narratives (Figure 1), with codes used to characterise what happened (incidents), reporter perceived reasons why the incident occurred (contributory factors), and the outcomes including type of harm.

Incidents were appraised and reclassified if required by one of the joint authors (the academic GP registrar) and the third author using the harm severity definitions from Cooper *et al* (2018),^29^ derived from a review of definitions of harm severity used internationally, and inclusive of psychological harms in primary care contexts. We reclassified incidents if they detailed harmful outcomes to patients or families in the free-text narratives. The third author double coded 20% of the 1000 reports. Consensus decisions informed one of the joint author’s subsequent coding decisions (the academic GP registrar). A Cohen’s Kappa statistic of 0.79 was calculated to assess concordance between this joint first author and the third author for harm severity. Subsequently, the third author and the other joint first author (the clinical academic community nurse) provided additional reviews in cases of uncertainty regarding reclassification to reach a consensus.

The Cooper *et al* classifications^29^ were grouped to mirror NRLS severity options used by the reporters to allow comparison (Box 2). During the study period, psychological harms were not explicitly included in NRLS harm definitions.^35^

**Box 2.** Definitions of harm used. Adapted from Cooper *et al.* ^29^ Example incidents for each harm severity can be found in Supplementary Box S1.^29^

Harm severity	Definition of harm severity
No harm/near miss	Incidents that occurred but caused no harm to the patient. This includes incidents that were mitigated before they caused harm
Mild harm	Incident in which: (a) patient was harmed, with mild and short-term impact, on physical, mental, or social functioning, that was expected to resolve in a few hours; (b) patient was harmed but required no or minimal intervention/treatment, for example, antiemetic, oral antibiotic, or repeat of a minor procedure such as vaccination or insertion of contraceptive implant; and/or (c) patient or their loved ones experienced transient emotional distress but no long-term consequences and incident report contains words, for example, angry, anxious, confused, distressed, frightened, frustrated, humiliated or upset, that might describe a feeling that occurs at the time of the incident but soon passes
Moderate harm	Incident in which: (a) patient was harmed, causing a medium-term impact on physical, mental, or social functioning that was expected to resolve in days; (b) patient required medical intervention in the form of treatment, for example, antibiotics or intravenous fluids; (c) patient required short-term hospitalisation for assessment and/or minor treatment in either an emergency department or a hospital ward; and/or (d) patient or their loved ones experienced psychological difficulty of a more long-standing nature but not requiring formal treatment, for example, as indicated by evidence in the report of more long-standing anxiety, insomnia, or low mood
Severe harm	Incident in which: (a) patient was harmed, causing a major long-term or permanent impact on physical, mental, or social function or shortening of life-expectancy; (b) patient was harmed and required major medical or surgical intervention that, most often, was delivered in a hospital setting, for example, cardioversion, any major surgery; (c) patient was harmed and required prolonged hospitalisation or admission to a high dependency unit and/or intensive care unit; and/or (d) patient or their loved ones experienced enduring psychological difficulty that required specialist treatment, for example, as indicated in the report by evidence of chronic anxiety or depression or psychosis
Death	Incident in which, on the balance of probabilities, death of the patient was caused or brought forward in the short term by the incident
Insufficient details	Insufficient information about the incident to evaluate the severity of harm

### Thematic analysis

**Figure 2.**
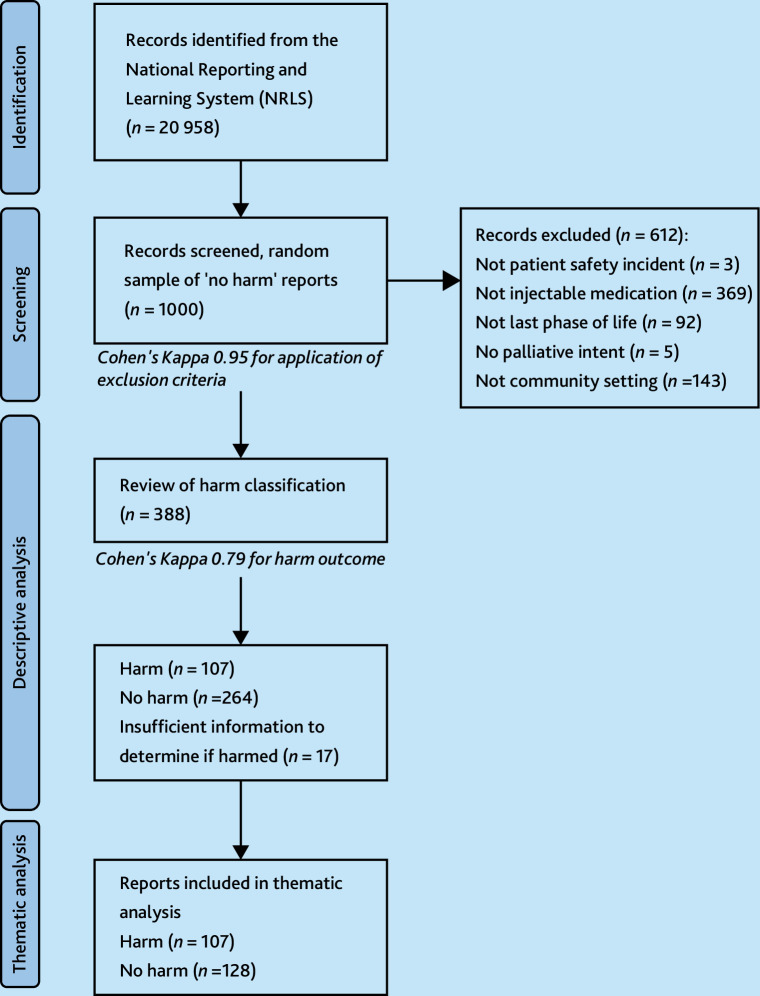
Overview of sample and study population identification steps.

Based on insights from the descriptive analysis, an inductive thematic analysis^36^ of the safety incident report narratives was undertaken using Nvivo 14 software. This analysis sought to understand the characteristics of reported ‘no harm’ incidents and how they differed from those reclassified as ‘harmful’; a sample of incidents was created based on the most frequent primary incident types within each group (Figure 2 and Supplementary Box S2). To ensure reflexivity, one of the joint authors (the academic GP trainee) discussed key analytical insights with the other joint first author, the senior author and the third author, and re-reviewed individual incident reports to ensure findings were firmly grounded in data.

Where appropriate, the reporting of this study complies with the Strengthening the Reporting of Observational Studies in Epidemiology (STROBE) statement.

## Public involvement

We met with our public and clinician advisory group to plan the study and discuss key findings. This group consists of four family caregivers with lived experience of end-of-life care and two practising community nurses. They provided valuable insights to guide the ‘implications for research and practice’ part of the discussion.

## Results

In total, 388 of the 1000 ‘no harm’ patient safety incidents were eligible and included in the descriptive analysis (Figure 2). Most incidents occurred in patients’ own homes (66%, *n* = 258/388), with 9% (*n* = 36/388) of incidents occurring during transfers between care settings and 7% (*n* = 28/388) in residential care homes. Patients were aged between 19 and 101 years (median age 75 years).

The majority of incidents related to medication prescribing and use (71%, *n* = 276/388), documentation (8%, *n* = 32/388), and treatments and procedures that did not involve medication (7%, *n* = 27/388) (Table 1).

**Table 1.** Primary incidents type categories involved (*N* = 388)

Primary incident^a^ type category	*n* (%)	Top two incident types for each category	*n* (%)
Medication prescribing and use	276 (71)	Medication not commenced in timely fashion	51 (18)
		Medication underdose	34 (12)
Documentation	32 (8)	Incorrect documentation or availability of medical records	17 (53)
		Care given but not documented	13 (41)
Treatment + procedure (not medication)	27 (7)	Insufficient treatment/care/monitoring given	16 (59)
		Incorrect process or procedure chosen	3 (11)
Diagnosis and assessment	18 (5)	Delay in assessment for care or care adjunct	11 (61)
		Premature discharge or poor discharge planning	6 (33)
Equipment	11 (3)	Issue related to the provision of therapeutic adjuncts	6 (55)
		Failure of equipment	2 (18)
Referrals	9 (2)	Referral not performed when indicated	2 (22)
		Incomplete/incorrect referral	2 (22)
Organisational processes	7 (2)	Difficulty accessing physician/healthcare professional	2 (29)
		Poor communication from secondary to primary care	1 (14)
Communication within healthcare	7 (2)	Poor communication between healthcare professionals	2 (29)
		Poor communication between healthcare professionals and patients	2 (29)
Investigation	1 (0.3)	Mislabelled sample/form	1 (100)

a Primary incidents are those occurring closest in time to incident outcomes.

Contributory factors were detailed in 95% (*n* = 367/388) of the incident narratives. Many incidents (55%, *n* = 215/388) had two or more contributory factors. These were frequently related to structural factors, including continuity of care (16%, *n* = 111/679), education and training (11%, *n* = 73/679), or related to staff factors such as mistakes and distractions (12%, *n* = 78/679), or inadequate skillset or knowledge (11%, *n* = 74/679).

### Incident outcomes

Incident outcomes were described in 87% (*n* = 337/388) of reports, with 189 of these reports describing staff or carers intervening to prevent or mitigate further harm. This commonly involved staff recognising prescription errors and ensuring that prescriptions were changed before the medication was required clinically. For example, a patient had been started on a syringe driver for pain control but required frequent additional doses. An excessive dose of ‘as required’ morphine was unintentionally prescribed by the out-of-hours service. The palliative care team reviewed the prescription chart and intervened to prevent the patient from coming to harm:

*‘* […] *Noticed the prescription error for morphine sulphate* […] *advised family* [controlled drug prescription] *form needs taking back to* [urgent care centre] *for re-writing* […] *No harm to patient and prescribing issue resolved at the time.*’ (Study ID 112; location: out-of-hours service base. Reported severity: no harm; remained as no harm.)

In total, 107 of the 388 reports contained harm and were reclassified using the Cooper *et al* classification system.^29^ This included 94 low harm incidents, 12 moderate harm incidents, and one resulting in hastened death:

*‘* […] *Community nurse reported administering* [antipsychotic drug] *15 mg* [subcutaneously] *to a patient as opposed to 1.5 mg according to prescription* […] *administered approximately Time 1. Realising error on reflection with colleague* [4 hours later] […] *Telephone call received from district nurse whilst on route to patient’s home to advise patient involved had died* […] *Required referring to crime scene investigation as advised by coroner*[…]*’* (Study ID 209; location: own home. Reported severity: no harm; reclassified as death. Type of harm: psychological and physical.)

### Comparison between no harm incidents and those reclassified as harmful

The incidents that were reclassified as resulting in harm differed from those that remained classified as ‘no harm’. They more frequently described issues with medication timeliness (26% [*n* = 28/107] versus 8% [*n* = 23/281]), or detailed medication overdoses (16% [*n* = 17/107] versus 5% [*n* = 13/281]). Incidents that remained classified as resulting in ‘no harm’ more frequently described lost medication (11% [*n* = 31/281] versus 0% [*n* = 0/107]) or medication not being prescribed despite being clinically indicated (8% [*n* = 22/281] versus 5% [*n* = 5/107]). Incorrect documentation was more frequently detailed in non-harmful incident narratives (11% [*n* = 30/281] versus 2% [*n* = 2/107]. For example, one patient that received medication via two syringe drivers had no further nurse visits booked after being discharged from the community nursing caseload by mistake:

*‘Patient had been visited by out-of-hours staff and they have discharged in error from* [community] *nurse caseload* […] *Admin reactivated patient referral, reinstated all of the schedules, and to reallocate to the nurses’ diaries for visit today*.’ (Study ID 97; location: community nursing base. Reported severity: no harm; remained as no harm.)

The most common contributory factors across all incidents reclassified as harmful involved continuity of care, the provision of care outside of normal working hours, and insufficiently skilled staff. Out-of-hours working was detailed as a contributing factor in 43% [*n* = 46/107] of harmful incidents, but in only 22% [*n* = 62/281] of no harm incidents. For example, out-of-hours working and organisational factors contributed to this harmful incident:

*‘Wife contacted* [community nurse] *to say medications had been collected and requested a* [community nurse] *to visit to administer* […] *Wife called again as no nurse had arrived* […] *Task sent to* [out-of-hours] *team to visit* […] *On arrival patient had secretions +++ and wife concerned that she had not seen a nurse yet* […] *Bank holiday period delay in prescription being dispensed by pharmacist* […]*’* (Study ID 23; location: own home. Reported severity: no harm; reclassified as low harm. Type of harm: psychological and physical.)

### Physical and psychological harms

Within the 107 incidents reclassified as harmful, 64 described physical harm only, 11 described psychological harm only, and 32 described both. Fifteen physical harm narratives detailed medication overdoses, 13 reported delays in giving medications for symptoms of pain and distress, and nine described medication underdoses. Incident narratives detailed several delays occurring, often as a chain of events exacerbated by insufficient staffing, especially during out-of-hours periods. For example, a patient had to go to hospital as a prescription chart could not be written in the community when needed:

*‘Called out during the night for pain relief and patient vomiting* […] *prescribed anti-emetic and pain relief* [as required] *for symptom control, however, frequency disallowed further doses during the night. Call to* [two sites] *regarding GP support and advice, no GP available at either site during the night* […] *Due to no GP available at either sites to prescribe and write up syringe driver, this patient was taken into accident and emergency at* [Hospital name] *to resolve this.’* (Study ID 86; location: own home. Reported severity: no harm; reclassified as moderate harm. Type of harm: physical.)

For the 43 incidents that detailed psychological harms, 29 detailed harms to family, eight to the patient and six to both. Within the 11 incidents that resulted only in psychological harm, four detailed communication breakdowns and two detailed delayed assessment of care or delays in administering medication. Emotional distress was the most common outcome:

*‘Patient discharged home yesterday for palliative care* […] *morphine dose was missing in the pink anticipatory prescribing sheet. The problem was rectified by GP surgery. Unacceptable delay in medication delivery and adding to family/patient distress*.’ (Study ID 317; location: transfer between care settings. Reported severity: no harm; reclassified as low harm. Type of harm: psychological.)

Incidents that contained both physical and psychological harms most frequently detailed delays in administering medication when required (44%, 14/32), insufficient treatment and monitoring (22%, *n* = 7/32), or delays in assessments (6%, *n* = 2/32). Within reports that described both psychological and physical harm, upset/emotional distress, pain, agitation, and delays in assessments or treatments were the most common outcomes.

### Thematic analysis

The main differences between the incident reports reclassified as harmful and originally classified ‘no harm’ incidents related to the quality of the report narrative, demonstrated more differences in opinion between caregivers and the family in the harmful reports, and contained evidence of differences in reporting culture.

#### Quality of report narrative

Incident reports that were reclassified as harmful tended to have longer, more detailed narratives and information about the impact of the incident on the patient and family members. The incidents that remained classified as ‘no harm’ provided brief accounts of when a process broke down, such as documentation errors or planned visits to administer drugs did not occur:

*‘I visited the patient with another staff nurse to re-prime syringe driver. When we counted the stock of midazolam ampules there was a discrepancy. The paperwork filled in on the previous visit by two other colleagues stated that there should be 32 left but we could only find 30.’* (Study ID 82; location: own home. Reported severity: no harm; remained as no harm.)

#### Differences in opinion between care providers

Narratives sometimes presented conflicting views about what constituted the most appropriate care. In the ‘no harm’ incidents this discordance was primarily between staff members, whereas harmful incidents more often detailed conflicting views among staff and family members. This discordance was because of differing accounts of when phone calls were placed or acted on, or because of differing views on the appropriateness of administering injectable medications:

*‘Patient’s son had administered injectable medication despite previous advice as his dad was unable to take oral medication* […] *Daughter expressed they were unhappy they had to wait for so long and he was unable to swallow his oral pain relief. He had eaten an egg sandwich for breakfast*.’ (Study ID 165; location: own home. Reported severity: no harm; reclassified as moderate harm. Type of harm: psychological and physical harm.)

#### Reporting culture

Reclassified harmful incidents were more commonly reported because a family member wanting to raise a complaint. ‘No harm’ incidents tended to be triggered by a retrospective staff realisation that established processes had not been followed, for example, when controlled drug vials were missing:

*‘When completing a stock check noted that morphine sulphate was not the correct amount recorded. Previous stock check states that there are 9 ampules but only 8 in the home.’* (Study ID 66; location: own home. Reported severity: no harm; remained as no harm.)

#### Misclassification of harm severity

In most reclassified harmful incidents, the reporter’s perception of harm seemed different to the definitions used in this paper: the reporter seemingly did not appraise that adverse psychological impact warranted a harmful categorisation, yet they took the time to detail the distress caused. Reports regarding significant delays in prescribing and administering medication to relieve suffering were often classified as ‘no harm’ by the reporter.

Incidents involving medication were sometimes retrospectively justified as causing no harm. These justifications included the drug being used for a different indication than prescribed, referring to suggested dose ranges in national prescribing guidance to warrant the overdose or underdose, or that the medication was out of date but still relieved the patient’s symptoms. An example of this was regarding a medication prescribed for nausea and vomiting being administered as a much higher unprescribed dose for agitation:

*‘On my arrival the paramedic had administered 25 mg levomepromazine as an injection* [for agitation] *but on the drug chart the levomepromazine was prescribed for nausea and vomiting and the* [as required] *dose was 2.5–10 mgs. Midazolam was the drug prescribed for anxiety and restlessness*.’ (Study ID 10; location: own home. Reported severity: no harm; reclassified as low harm. Type of harm: physical.)

## Discussion

### Summary

Our study identified that physical harms and psychological harms are described within 28% (*n* = 107/388) of apparent ‘no harm’ patient safety incidents. We found that the incident reports reclassified as harmful and the true ‘no harm’ incidents differed in terms of the quality of the report, the harmful incidents contained more conflicting views of what constituted appropriate care between professionals and family members, and notable variability in perceptions of what constitutes harm. We found harmful incidents were being misclassified as ‘no harm’ owing to differing perceptions of harm definitions and medication errors being retrospectively explained and justified.

### Strengths and limitations

The random sampling of nationally reported ‘no harm’ incidents reflect the real-world reporting in a large healthcare system. Two researchers reviewed inclusion and harm severity, conferring with a third researcher in the event of any doubt, to ensure rigour and consensus of our classification. The mixed-methods approach sheds light on how different factors interact and contribute to reporters’ perceptions of harm and how reported harm classifications can conflict with recorded insights gained from patients and families.

This study is limited partially because of the small amount of free-text information in many of the incident reports. Incident reports are socially constructed narratives,^37^ shaped by cultural beliefs and professional norms. Although we utilise taxonomies and classification systems, reports are open to subjective interpretation and often provided limited information on perceived harms in emotive situations.^21^ These reports are written by healthcare professionals often just after the incident with limited information, especially in relation to longevity of impact and therefore severity of harm.

As we purposefully used Cooper *et al*’s Harm Severity Classification System (2018)^29^ rather than NHS England definitions available at the time the incidents were reported,^35^ we expect some of the reports were classified as ‘no harm’ as these NHS England definitions did not include psychological harm at the time. NHS England have now introduced definitions of psychological harms to help guide reporters.^32^

### Comparison with existing literature

The types of recurrent incidents we identified are consistent with previous research on primary care out-of-hours palliative care patient safety incidents^24^ that defined four common areas in which palliative care incidents occurred: medication provision, timely care, information transfer, and treatment provision. A recent study investigating syringe driver-related incidents also identified that free-text narratives described psychological distress to patients and family members that were infrequently reflected in the reported harm classifications.^30^ Our work builds on this by assessing the frequency of physical and psychological harm to patients and family carers and exploring the themes relating to this distress.

Harms tend to be underreported by voluntary incident reporting systems both within palliative care^24,26,30^ and more generally.^38–40^ Staff can become desensitised to delays in visiting to administer symptom control medication and stop noticing such problems, or avoid reporting incidents or downgrade the severity because of fear of a blame culture, especially if reports go through their manager.^21^ Although the narratives in this paper were often insufficient to determine why reporters misclassified harm as ‘no harm’, this paper lays the groundwork for investigating this further through qualitative research with healthcare staff and families.

### Implications for research and practice

Healthcare systems need to develop better ways to report and learn from reported patient safety incidents, with a focus on patient and family expressed experiences of suboptimal care. Healthcare teams should consider non-physical consequences of patient safety incidents, including psychological outcomes. Importantly, patient safety incidents, especially within palliative care, should consider the patient and their caregiver/family members as a care unit. Some healthcare systems allow reporting by patients or families^41–43^ but this needs to be widely encouraged and publicly advertised. Giving patients and family/caregivers a voice in reporting the impact of patient safety incidents will provide a more holistic understanding of events and harms.

NHS England’s recent guidance on recording patient safety events includes detailing psychological harm separately to physical harm.^32^ This is valuable and we suggest worked examples, such as those seen within Cooper *et al*’s Harm Severity Classification System table,^29^ would increase the accessibility of this guidance. In addition, training on harm severity classification should be available to those that are expected to report patient safety incidents. We suggest that a typology should be developed to better capture psychological harms when reporting incidents in palliative care, including not only the timescale of harm to the patient and the treatment required, but also how this had an impact on the families and how this altered grief and bereavement for those involved.

This study found that 28% (*n* = 107/388) of ‘no harm’ incidents contained harm. Other research has highlighted that reporters are also misclassifying harmful incidents.^30^ Further research is warranted to investigate how accurately harm severity is being classified.

Artificial intelligence (AI) systems are being developed to support patient safety efforts,^44–46^ including to identify and classify incidents across specified patient safety categories,^47–50^ and to assist the reporting of incidents.^51^ These AI systems could be utilised to support reporters in classifying incidents or to provide guidance about additional information required to support judgements about the severity of impact. However, this is evolving technology and ethical consideration is needed about what may happen to sensitive patient safety information if it is submitted into large language models.

Accurately reporting harm alone is insufficient and reports must be utilised as a trigger to investigate events, identify and act on learning, and support a safety culture that is inclusive of learning from psychological harms and the concerns of patients, families, and caregivers.

## Data sharing

We are unable to share primary data. These data are held by NHS England and applications for using NRLS data are decided on a case-by-case basis.
